# Barriers to sexual and reproductive care among cisgender, heterosexual and LGBTQIA + adolescents in the border region: provider and adolescent perspectives

**DOI:** 10.1186/s12978-022-01394-x

**Published:** 2022-04-12

**Authors:** Randolph D. Hubach, Rebecca Zipfel, Fatima A. Muñoz, Ilana Brongiel, Annabella Narvarte, Argentina E. Servin

**Affiliations:** 1 College of Health and Human Sciences, Purdue University, Matthews Hall, 214A, West Lafayette, IN 47907 USA; 2 San Ysidro Health, 333 H Street, Suite 2080, Chula Vista, CA 91910 USA; 3 School of Medicine, Division of Infectious Diseases and Global Public Health, University of California, San Diego, 9500 Gilman Drive MC0507, La Jolla, CA 92093-0507 USA; 4 Department of Medicine, University of California, San Diego, 9500 Gilman Drive MC0507, La Jolla, CA 92093-0507 USA

**Keywords:** Adolescent health, Sexual and reproductive health, LGBTQIA +, Access to sexual and reproductive health, U.S.-Mexico border region, Adolescentes, Salud sexual y reproductiva, LGBTQIA +, Acceso a la salud sexual y reproductiva, Región fronteriza México-Estados Unidos

## Abstract

**Introduction:**

The United States (U.S.) has higher rates of sexually transmitted infections (STIs) and adolescent pregnancy than most other industrialized countries. Furthermore, health disparities persist among racial and ethnic minority adolescents (e.g., African American and Latinx) and in counties located along the U.S.–Mexico border region—they demonstrate the highest rates of STIs and unintended pregnancy among adolescents.

**Methods:**

Qualitative data were collected as part of formative research for the development of a mobile app that provides gender-inclusive sexual education to adolescents living in the U.S.—Mexico border region. From August 2019 to March 2020, the study team conducted 11 in-depth interviews with healthcare providers and three focus groups with cisgender, heterosexual, and SGM adolescents ages 15–18 (n = 20).

**Results:**

Providers and adolescents reported similar barriers to accessing SRH in this region such as transportation, lack of insurance and cost of services or accessing services without their parent’s knowledge. However, providers shared that some adolescents in this region face extreme poverty, family separation (i.e., parent has been deported), have a mixed family legal status or are binational and have to travel every day from Mexico to the U.S. for school. These challenges further limit their ability to access SRH.

**Conclusions:**

Adolescents in the U.S.-Mexico border region face unique economic and social challenges that further limit their access to SRH care, making them uniquely vulnerable to STIs and unintended pregnancy. The prototype of the app was developed based on the needs expressed by providers and adolescents, including providing comprehensive Sex Ed and mapping of free comprehensive and confidencial SRH services available in the region and is being pilot tested. Our findings provide further evidence for the need for interventions and service delivery, programs tailored for residents in the border region.

## Plain language summary

The United States (U.S.) has higher rates of sexually transmitted infections (STIs) and adolescent pregnancy than most other industrialized countries. Furthermore, health disparities persist among racial and ethnic minority adolescents (e.g., African American and Latinx) and in counties located along the U.S.–Mexico border region—they demonstrate the highest rates of STIs and unintended pregnancy among adolescents. A study was conducted as part of formative research for the development of a mobile app that provides gender-inclusive sexual education to adolescents living in the U.S.—Mexico border region. From August 2019 to March 2020, the study team conducted 11 in-depth interviews with healthcare providers and three focus groups with cisgender, heterosexual, and SGM adolescents ages 15–18 (n = 20). Providers and adolescents reported similar difficulties accessing care such as transportation, lack of insurance and cost of services or accessing services without their parent’s knowledge. However, providers shared that some adolescents in this region face extreme poverty, family separation (i.e., parent has been deported), have a mixed family legal status or are binational and have to travel every day from Mexico to the U.S. for school. It is evident that adolescents in the U.S.-Mexico border region face unique challenges that further limit their access to care, making them uniquely vulnerable to STIs and unintended pregnancy. The prototype of the app was developed based on the needs expressed by participants and is being pilot tested. Our findings provide further evidence for the need for service delivery and programs tailored for residents in the border region.

## Resumen

**Introducción:**

Estados Unidos (EE. UU.) tiene tasas más altas de enfermedades de transmisión sexual (ETS) y embarazo en adolescentes comparado a otros países industrializados. Además, las disparidades de salud persisten entre los adolescentes que son minorías (por ejemplo, Afroamericanos y Latinos) y en los condados ubicados a lo largo de la región fronteriza de EE. UU. y México, donde se muestran las tasas más altas de ETS y embarazos no deseados en adolescentes.

**Métodos:**

Se recolectaron datos cualitativos como parte de una investigación formativa para el desarrollo de una aplicación móvil que brinda educación sexual a adolescentes que viven en la región fronteriza. De agosto del 2019 a marzo del 2020, se llevaron acabo 11 entrevistas a profundidad con proveedores de salud y tres grupos focales con adolescentes cisgénero, heterosexuales y LGBTQIA + de 15 a 18 años (n = 20).

**Resultados:**

Los proveedores y los adolescentes informaron barreras similares para acceder a los servicios de salud sexual y reproductiva (SSR) como el transporte, la falta de seguro médico, el costo de los servicios y acceder a los servicios sin el conocimiento de sus padres. Además, los proveedores compartieron que algunos adolescentes enfrentan pobreza extrema, separación familiar (por ejemplo, el padre ha sido deportado), tienen un estatus legal familiar mixto o son binacionales y tienen que viajar todos los días de México a los EE. UU. para ir a la escuela.

**Conclusiones:**

Los adolescentes en la región fronteriza de EE. UU. y México enfrentan desafíos económicos y sociales únicos que limitan aún más su acceso a SSR, lo que los hace especialmente vulnerables a las ETS y a los embarazos no deseados. El prototipo de la aplicación fue desarrollado en base a las necesidades expresadas por los proveedores y los adolescentes, como educación sexual integral y mapeo de los servicios de SSR gratuitos y confidenciales disponibles en la región, y se esta llevando acabo la prueba piloto. Nuestros hallazgos evidencian la necesidad de intervenciones y programas de SSR que se adapten a las necesidades únicas que enfrentan los adolescentes en esta región.

## Background

Within the United States (U.S.), cases of sexually transmitted infections (STIs) have continued to rise in recent years [1]. One geographic area with significant STI burden is the border region between the U.S. and Mexico, where the rates for STI have continued to increase in the past five years [2]. There was a 9.6% increase in cases of chlamydia and a 18% increase in cases of gonorrhea from 2017 to 2018 in the previous 12-month period [3–5]. Further, among ethnicities aged under 20 years, African American (AA) and Latinx populations had the highest rates of chlamydia and gonorrhea [3–5]. The region along the U.S.-Mexico border experiences high rates of poverty, low rates of medical insurance, low rates of health literacy exacerbated by language issues, and minimal access to healthcare providers [6–9]. Increasing access to sexual and reproductive (SRH) services is a key priority of the Health Border 2020 initiative to address SRH disparities within these underserved and under resourced communities; however, few studies have explored the SRH service needs and preferences among adolescents living in the border region [10–12].

While individual-level determinants, such as high-risk behaviors, contribute to disease transmission and acquisition risk, it is widely accepted that social barriers to STI prevention and control efforts also contribute to infectious disease prevalence. The accessibility of SRH services is influenced by a complex set of factors related to adolescent’s awareness of services, socio-cultural norms regarding sexual activity of adolescents, availability of services, costs of using the services and the quality the services they provide. These factors are exacerbated by the lack of comprehensive sexual education in the U.S. wherein information shared in schools is highly variable depending on the state and sometimes the city in which a student lives. The result of this variability is that some students receive little to no pertinent information on sexual health and sexuality which can lead to increased sexual risk-taking behaviors (e.g., condomless sex, multiple sexual partners, anal sex) among adolescents and emergent adults [13–18]. For sexual and gender minority (SGM) adolescents, sex education tends to be an experience that leaves them feeling invisible [19, 20]. This invisibility can reinforce feelings of isolation and marginalization, leading to higher rates of depression, suicidal ideation, increased number of sexual partners, and higher rates of pregnancy [21–24].

Adolescents often find themselves not understanding their rights to accessing SRH services, including privacy and confidentiality, or find themselves unable to navigate care systems. This can ultimately impact adolescents’ comfortability once in a clinical setting [25, 26]. The desire for privacy and confidentiality may hinder SRH discussions with routine medical providers out of caution that doing so could change the provider’s attitudes toward them and impact the quality of care they receive. By choosing not to disclose sexual behavior or sexual orientation information, pertinent knowledge and care are unintentionally withheld from adolescent patients, including information about primary and secondary prevention methods.

Improving linkage to affirming SRH services for adolescents and young adults has been demonstrated to decrease STI transmission, unintended or unwanted pregnancy, and increase the uptake of preventative behaviors (e.g., condom use, Pre-Exposure Prophylaxis) [27–29]. Thus, the goal of this study was to explore the barriers for access and linkage into care experienced by cisgender, heterosexual, and LGBTQIA + adolescents as well as SRH providers that work with adolescents in clinic sites located in high schools near the U.S.–Mexico border region in order to inform a mobile-app intervention to improve access and linkage to SRH care.

## Methods

### Study setting

This study took place in the Sweetwater Union High School District; it was selected because it is the largest secondary school district in California and is located in the U.S.-Mexico border region [30]. Approximately 87% of students belong to an ethnic minority group and ≥ 58.5% qualify for a free/reduced lunch [31]. This region also has higher rates of STIs, unintended pregnancy, and illicit drug use than the national average [3–5, 32, 33]. Our community partner has been working with this school district for the past two years and has successfully established school-based clinics across three high schools in this district.

### Study design

Utilizing community-engaged research approaches, the principal investigator (PI) conducted in-depth interviews (n = 11) with social and healthcare providers, administrators, local community leaders from both clinics and non-government organizations (NGOs) that work with heterosexual, cisgender, and LGBTQIA + adolescents in the proposed school district [34, 35]. Further, focus groups (n = 3; 20) were conducted with cisgender, heterosexual, LGBTQIA + adolescents seeking SRH services at the school-based clinics as well as a sexual health clinic located in the school district that offeres access to free services to adolescents and young adults, to assess adolescent needs, identify barriers to accessing care, and key services available.

### Recruitment

For the in-depth interviews (IDI) with providers, utilizing a purposive sampling approach, potential participants received an email from the study team inviting them to participate in an in-depth interview regarding their work with adolescents, highlighting that the interview was completely voluntary and confidential, and including information on the best way to reach the research team to schedule an interview based on their availability. Eligibility criteria included: (i) being an SRH adolescent provider, *promotores*, health educator, administrator, community leaders/advocates for adolescent health in an organization located in the Sweetwater Union High School District; (ii) age > 18 years-old; (iii) have provided services and/or work directly with cisgender, heterosexual and/or LGBTQIA + adolescents at least once a month or involved in delivery or administration of SRH services for adolescents; (iv) able to provide voluntary informed consent; (v) literate in English or Spanish. All participants provided voluntary written informed consent when they arrived to the IDI and received a $20 gift card for their participation. Additionally, focus groups (FGs) were also conducted with cisgender, heterosexual and LGBTQIA + adolescents ages 15–18 years of age seeking confidential SRH services at a teen clinic and the school-based clinics located in the service area. Inclusion criteria for the FGs included: (i) currently enrolled as a student in a high-school located in the Sweetwater Union High School District; (ii) age 15–18 years old; (iii) self-identify as cisgender, heterosexual and/or LGBTQIA + ; (iv) literate in English or Spanish; (v) able to provide voluntary informed consent. We included both adolescents who are sexually and not sexually active. Local female research staff approached adolescents in the waiting rooms of the participating clinics (e.g., the teen clinic or the school-based clinic) and provided them with a study recruitment card. The recruitment card had a unique ID, explained the purpose of the study and encouraged those interested in participating to text the listed phone number with the words “Interested?” or “te interesa?” and the unique ID. The phone number was a Google Voice number set-up and monitored by the study research staff that masked the direct line’s outgoing and incoming phone numbers (to protect the participants privacy). After potential participants texted the number, the study research staff confirmed with the client that they met the eligibility criteria through text messages. If the client was eligible, they were asked through messaging if they were interested in participating in an upcoming FG discussion. If they confirm their interest, the research staff arranged for them to attend an upcoming FG discussion at the teen clinic through text messaging. At all points of communication, staff emphasized that participation was completely voluntary and that their access to services at the clinics would in no way be impacted by their decision to participate or not, or the nature of their participation. All participants provided voluntary written informed consent when they arrived to the FG session and received a $20 gift card for their participation in the FG. The study was approved by the Institutional Review Boards at the University of California, San Diego and San Ysidro Health’s Ad hoc IRB review committee.

### Data collection

Female research assistants and the PI conducted a total of eleven IDIs in English. The interviews were audio-taped and lasted approximately 60 min. Questions explored issues related to the SRH needs of adolescents based on the providers experiences, barriers to accessing services, services available at their organization and other nearby locations that service adolescents. Similarly, female research assistants and the PI conducted a total of 3 FGs (n = 20; 6–7 participants per group) with cisgender, heterosexual, and LGBTQIA + adolescents. FGs were audio-taped (identified using only a study-unique identification number and pseudonyms) and lasted 60–90 min. The questions followed a semi-structured open-ended guide informed by our previous work with medically underserved adolescents and explored themes related to.

adolescent needs, barriers to accessing care, prevention programming, and key services that could be incorporated to the app to increase linkage into care [36–38].

### Data analysis

All interviews and FGs were conducted in English. Transcripts from the interviews and focus groups were transcribed verbatim by the research team. Qualitative analysis was led by the PI in conjunction with three members of the research team. The research team systematically read through transcripts, engaged in open line-by-line coding and constructed a coding scheme based on the content of the transcripts which was iteratively revised until the research team reached consensus. Transcripts were coded in ATLAS.ti version 6.2 to group, label, and describe intersections between emergent themes related to the SRH need of cisgender, heterosexual, and LGBTQIA + adolescents, sexual education (Sex Ed) content (i.e., sexuality, consent, gender norms, birth control, HIV/STIs, myths, etc.), barriers to accessing care, key services available that can be incorporated to the app to increase linkage into care, feasibility and acceptability of using a feature for linkage into care for adolescents [39, 40]. Thematic saturation as directly and broadly related to the aforementioned research questions, was reached with the eleventh IDI and the third FG. Using the final coding scheme, inter-coder reliability was assessed and achieved greater than 80% consistency between the coders. This analysis adopted deductive and inductive perspectives in which participants’ language and experiences were used to interpret our research questions [41]. For ethical and confidentiality purposes, names of participants reported in the results have been changed.

## Results

### Participant characteristics

Among the twenty cisgender, heterosexual, LGBTQIA + adolescents (n = 20) that participated in the FGs, the mean age was 16.1 years and all participant where currently enrolled in high school. Thirty percent (n = 6) identified as LGBTQIA + and 70% (n = 14) as cisgender and/or heterosexual (Table 1). Among the social and health care providers that participated in the IDIs (n = 11), 36.3% (n = 4) identified as male, 54.4% (n = 6) as female and 9.0% (1) as transgender. We had a good representation of different services providers including school-based physicians (n = 2), social service providers (n = 2), school-based nurse (n = 1), teacher (n = 1), licensed marriage and family therapist (n = 2), peer-health educator (n = 2) and a community health specialist (n = 1) and 66.6% (n = 8) had been working with adolescents for 5 or more years (Table 2).

**Table 1:** Characteristics of adolescents (N = 20) seeking sexual and reproductive health services in the U.S.—Mexico border region

Variable	N = 20 (100%)
Age (mean)	16.1
Gender	
Male	4 (20%)
Female	16 (80%)
Education	
Currently enrolled in high school	20 (100%)
Speaks another language other than English at home	16 (80%)
Self-identify as LGBTQIA +	6 (30%)
Self-identify as cisgender and/or heterosexual	14 (70%)

**Table 2:** Characteristics of social and healthcare providers in the U.S.—Mexico border region serving adolescents (N = 11)

Variable	N = 11 (100%)
Age	
18–24 years old	2 (22.2%)
25–34 years old	2 (22.2%)
25–44 years old	2 (22.2%)
45–54 years old	5 (45.5%)
Sex	
Male	4 (36.3%)
Female	6 (54.4%)
Transgender	1 (9.0%)
Race/Ethnicity	
White	5 (45.5%)
Asian	1 (9.0%)
Hispanic or Latinx	5 (45.5%)
Occupation	
School-based healthcare provider (e.g., physician)	2 (22.2%)
Social services provider	2 (22.2%)
School-based nurse	1 (9.0%)
Teacher	1 (9.0%)
Licensed marriage and Family Therapist (LMFT)	2 (22.2%)
Peer-health educator	2 (22.2%)
Community Health Specialist	1 (9.0%)
Number of years working with adolescents	
< 1 year	1 (9.0%)
1—5 years	4 (36%)
5—10 years	2 (22.2%)
10—15 years	2 (22.2%)
> 15 years	2 (22.2%)

## Physical barriers to accessing SRH

According to providers in the area, SRH services are available but not always readily accessible to adolescents. Nine out of the eleven providers that participated (81%) listed transportation as the dominant physical barrier that makes it challenging for adolescents to access services. When a school-based provider refers a student to services in the area (because they do not have the resources there), the student is responsible for their own transportation to and from the clinic.

“*Transportation is one [barrier], because if we do refer someone to the community health center, they’re responsible for providing their own transportation. Even though they can leave campus without their parent’s knowledge and go to the health center, they still have to get there. Unless they’re savvy and know how to use the bus system or have someone take them, it’s pretty difficult to get here.” (IDI, Health and Physical Education Teacher, Male )*

*“… one of the main barriers was location of wherever they’re getting their services. Uhm, so our clinic was near schools, like it was still hard for them to walk, like it wasn’t next door to a school or anything, it was like a mile away which is, you know, like if they don’t have a car, then it’s hard for them to access, so definitely transportation.” (IDI, Peer-Health Educator, Male)*

Given the close proximity of this school district to the U.S.-Mexico border, providers reported serving a large bi-national adolescent population. Providers reported that approximately 30–50% of students are separated from their nuclear family in order to attend schools in the U.S., while other students cross the border on a daily basis. Other students experience homelessness or housing insecurity. Providers described how instability of the home or disrupted living situations contributed to physical barriers in adolescents seeking and maintaining proper SRH care.

*“I think the biggest barrier in the population we have is that they are dealing with a lot of chaos in their families. Be it financial problems, be it parents being separated due to being deported” (IDI, Youth Enhancement Services Provider, Male)*

*“It’s not just as easy as I don’t know where to go? It’s how am I gonna get there? If I have a kid, who’s gonna watch my kid, how am I going to take off work when I don’t get paid that much anyway because I have to pay this money so that my parents and we can still live here” (IDI, Director of Education and Engagement, Female)*

Adolescents collectively agreed that transportation was a factor in attaining SRH services. Some of the concerns included not being able to rely on a relative or older adult without their parent’s knowledge and the costs of public transportation being a barrier for most adolescents.

*“There’s no transportation and that’s a big problem and again… like what Beet said [the other participant in the FG], students with conservative parents, they have a hard time going behind their backs or - well, that sounds bad, or just accessing health services without permission.” (FG participant, Female, 16 years old)*

## Insurance and cost of services

Providers recognized that a majority of the population served in the school district area are low-income or in need of an insurance plan. Families are financially struggling to afford basic necessities, i.e., rent, and oftentimes adolescents find themselves in a position of responsibility to support their family.

*“Poverty…putting food on the table and a roof over their heads is a priority or dealing with all of the chaos that’s happening in their lives is a priority so this may not be number one. We get more probably no-shows or non-returning to first services” (IDI, Youth Enhancement Services Provider, Male)*

*“I think part of it is cost, part of it is going to the store and buying them [condoms]. That could be a barrier” (IDI, Health and Physical Education Teacher, Male)*

Some of the school-based providers stated that approximately 90% of the students in schools in the district are living below the federal poverty line. According to providers, a large proportion of families qualify for free or low-cost insurance, but do not receive the assistance needed to properly enroll. When providers serve families that have health insurance plans, they often find that a lack of knowledge or awareness prevents adolescents from regularly accessing the SRH services they need.

*“Some of the families do have insurance, a small percentage … but when you look at their charts, they’re not very frequent visitors to clinics, teens in general don’t access care unless they have a medical issue very often, they’re generally a pretty healthy population so they might have insurance and they’re often not sure what they have” (IDI, Associate Program Director, Female)*

*“… they think they’re going to have to pay for it, they don’t understand that insurance will cover the majority, or 100% pretty much, of the services...” (IDI—Director of Education and Engagement, Female)*

*“What I’ve seen is a lot of students with health insurance…they have to go with their parent to those places and they don’t know how to navigate the confidential services.” (IDI, Associate Program Director, Female)*

Providers also noted that adolescents often do not know what type of insurance plan their family is enrolled in or are not aware that they can still use insurance coverage to access services without their parent’s knowledge. Majority of adolescents in the area who access SRH at the clinics qualify for Family Pact (FPACT). However, most of them were unaware and would typically enroll when they would come to the clinic for care.

*“So primarily for our adolescent clinic, we almost exclusively use FPACT… that would basically make sure they don’t have to pay anything...I know that sometimes they did use their private insurance, but they would have to have the form that says “okay, this shouldn’t come on the billing because it’s confidential services” Uhm, so kind of make things easier, we would just enroll them into Family Pact” (IDI, Health Educator, Female )*

Adolescents agreed that no insurance or low quality of insurance (e.g., limited coverage) makes SRH services such as birth control and hormone replacement therapy (HRT) inaccessible. Additionally, they acknowledged the difficulties in navigating insurance to cover the cost of SRH services without a parent’s knowledge.

*“It’s hard for people of economic minority, to buy birth control and things like that because birth control is expensive as is and especially if you don’t have insurance, and if they don’t have insurance, I feel like these people would rather spend their money on other things like paying their bills or food, than on reproductive health.” (FG participant, Female, 15 years old)*

*“Teenagers don’t have access to their own insurance cards. So, I can’t just go to the doctor myself, with my own insurance card and do my own stuff without my parent knowing… They [teenagers] can’t do it on their own, because they don’t know how… like they don’t want anything getting back to their parent, basically.” (FG participant, Female, 17 years old)*

## Parental influence on SRH services uptake

Many adolescents are unable to talk to their parents about issues related to SRH as they fear they will disapprove. Accessing services without their parent’s knowledge is necessary, however, many spoke about fears that their parents would find out or were not aware that they could access SRH without parental permission. Every provider described parental influence or permission to be a common barrier. When serving adolescent populations, they are frequently asked questions regarding the confidentiality of their visit.

*“They think that their parents have to be involved somehow, so one of the main questions that we get when folks reach out for HIV testing, before anything else, is “Do my parents have to know?” (IDI—Youth Services Department Manager, Transgender)*

Similarly, perceptions of what parents may allow or not allow often dictated services or prevention methods adolescents might utilize.

*“A lot of students don’t want to use contraception because parents aren’t supportive of that…” (IDI, School-based Physician, Female)*

*“We’ve definitely had a lot of students who haven’t gotten the care they need because they did not want to ask their parents for permission….” (IDI, School-based Nurse, Female)*

*“I’ve noticed that some of the parents usually say, “you don’t need that” or “you’re not at that age to get any of that”, and they are clearly at that age where they want to know what it’s like and they need to know about protection and everything…” (IDI, Health Educator, Male)*

## Cultural and political climate

Providers reported that some of their patients do not feel comfortable applying for government programs that are low cost or no cost due to immigration status and fear based on the current political climate. This created an additional barrier for adolescents to access care.

*“A newer barrier… is a lot of concern around even enrolling in FPACT because immigration status. At one of my other sites… she [client] was in the process of an immigration … and she said, “ I don’t want to get any government [support], is FPACT a government program?” And I said yes, it’s part of Medi-Cal and she said, “I don’t want it, I’ll just pay for it.” And that’s not really realistic to pay for these services, especially if you’re a teen.” (IDI, Associate Program Director, Female)*

*“… with the concerns around public charge, there is fear in the family because there’s mixed status [immigration status] in the family that’s going to affect someone. (IDI, School-based Physician, Female)*

Likewise, participants mentioned there is some fear or stigma in general when engaging in SRH for the first time. Participants noted it was necessary to create a space where adolescents feel comfortable to help eliminate this initial fear.

*“So, some of our staff will go out to the clinics and make sure it’s youth- friendly. Getting rid of the initial fear of walking through the door is a big deal.” (IDI, School-based Community Service Provider, Female)*

*“I don’t think they feel comfortable enough to come up to their teacher. So I definitely think there’s a better way to get information. Because I do get students, but I think there’s a lot more students that are just afraid to ask.” (IDI, Health and Physical Education Teacher, Male)*

## Stigma experienced by LGBTQIA + adolescents

Not surprisingly, providers mentioned that family rejection and peer victimization were common among LGBTQIA + adolescents. The disclosure of their gender or sexual identity to their family members caused significant interpersonal problems among adolescents and their families such as being kicked out of their homes and becoming homeless.

*“Particularly for LGBTQ kids, because of the stigma they face at home, they get kicked out much earlier than their straight and cisgender counterparts, so we have a lot of folks, like who need a place to stay, need a safe house to move into. So, a lot of things we do too, is linkage to other agencies that have housing resources.” (IDI, Youth Services Department Manager, Transgender)*

“… *certainly, there’s a group on campus, like an affinity group for LGBTQIA*+ *students… and the students that I’ve talked to, I ask around safety, and bullying and all of those things and none of them have identified that as a particular issue, but I think that there’s definitely a bias towards heteronormative services. I do have one transgender patient at one of the schools, that I’ve been trying to get plugged in for transgender stuff, but it’s been incredibly challenging.”* (*IDI, School-based Physician, Female*)

Further, LGBTQIA + adolescents have additional barriers to accessing SRH care in this region. They find it difficult to share access specific LGBTQIA + care as some clinicians are not well trained in addressing the concerns of members of this community.

**“***… If you are transgender and non-binary, walking into any agency even if it’s a healthcare organization, you’re automatically assuming that people are not gonna understand what transgender and non-binary is, they’re not gonna have a place for you to explain what your pronouns and what your chosen name is. Uhm, so you are automatically putting yourself in a situation where you’re either going to be mentally stressed out or physically in danger.” (IDI, Youth Services Department Manager, Transgender*)

## Discussion

Numerous reports have highlighted SRH for adolescents living near the U.S.-Mexico border as a priority target area in order to reduce health inequities and promote healthy outcomes in this community [12, 42]. This includes a myriad of barriers that adolescents have to navigate in order to access SRH services. Barriers include the physical difficulty of reaching the clinic if not on school grounds, as many adolescents do not have their own transportation and public transport is unreliable and expensive for them. This is consistent with previous research that has identified transportation and location as common barriers that reduce adolescents’ access to SRH care in the U.S. and elsewhere [43–45]. Issues with insurance include a lack of sufficient coverage and the inability to pay for services due to the high rate of adolescents living below the federal poverty line. Even for adolescents who do have coverage, many are unaware of what it covers, or do not want their parents to find out that they are accessing SRH services as they fear they will disapprove. SRH qualifies as a confidential service and parental consent is not required, however many adolescents do not know this or how to navigate the system to ensure confidentiality is maintained. Additional fears relate to social stigma around SRH as well as the current political climate, as some of the adolescents lack documentation or are worried due to the immigration status of a family member and are therefore reluctant to sign up to any governmental program.

Parental opinions, including the inability to discuss SRH with parents and concerns over the confidentiality of services, is one of the key barriers that adolescent populations face —especially when using their family insurance for SRH if their parents disapprove [46]. Studies have documented that over 40% of adolescents are already sexually active prior to having a conversation with their parents about SRH issues and parental beliefs and misconceptions around the Human papillomavirus (HPV) vaccine promoting sexual activity decreases the number of Latinx adolescents in Southern California who get this SRH preventative care [47, 48]. Concurrently, adolescents continue to navigate social and cultural norms that stigmatize SRH and accessing care wherein adolescents fear the unknown in terms of physically going to a clinic as well as the apprehension of assumptions, judgment and discrimination from providers and other clinic staff [14, 28, 46]. Such norms are exacerbated among LGBTQIA + adolescents as they navigate systems entrenched within heteronormative perspectives related to SRH [49]. These findings highlight the need for interventions to increase knowledge and linkage of adolescents to SRH in a way that can be discrete and easy for them to use while reframing access to SRH services as a normative experience.

Creating a “youth-friendly” environment in adolescent SRH clinics has been found to be essential, and fits with what we found in our research. For example, ensuring staff are trained to communicate with adolescents in a non-judgmental way, that they use the correct pronoun, maintain confidentiality, have cultural sensitivity, and provide the option of walk-in appointments, have all been shown to help remove some of the barriers for adolescents wanting to access SRH services [14, 21, 26, 29]. This is important for all adolescents, but especially for those who identify as LGBTQIA + and those living near the U.S.-Mexico border, as they are already burdened by environmental factors, thus making the need for trauma-informed care and the creation of a safe and non-judgmental space even more paramount in order for them to feel comfortable and willing to access SRH services.

Furthermore, the adolescents in our study, as well as others, have shown as overwhelming desire for access to more information on information on SRH [13, 14, 50]. Despite the introduction of the California Healthy Youth Act in 2016, that mandates all California schools to provide integrated, comprehensive, accurate and inclusive Sex Ed at least once in middle school and once in high school, the actual delivery of this education differs greatly between districts. Parents are able to opt their child out of these classes and San Diego in particular has faced a lot of protest against the inclusion of this information in the educational curriculum [14–17, 51]. Leveraging mobile health (mHealth) technology to provide adolescents with additional SRH information is one promising approach [50, 52]. There are various benefits to turning to mHealth to enhance sex education, including developing a sense of agency, anonymity, and the ability connect with others.

### Strengths and limitations

This current study and the conclusions made are not without some limitations. As our purpose was to inductively explore the experiences of providers and adolescents within a specific region of the U.S.-Mexico border, we cannot assume our findings necessarily generalize to other adolescent populations across the U.S. In addition, adolescents were recruited from a healthcare and school setting in which they were seeking care and thus, may not represent the most vulnerable adolescents within the region. Nevertheless, these limitations should be considered with the study strengths. We engaged both provider and adolescents to triangulate findings related to barriers to SRH services among an underserved and under investigated area along the U.S.-Mexico border. These results emphasize the necessity to develop affirming systems of SRH care and accompanying resources to enhance adolescent SRH knowledge related to prevention, screening, and navigating care systems.

## Conclusions

Adolescents in the U.S.-Mexico border region face unique economic and social challenges that further limit their access to SRH care, making them uniquely vulnerable to STIs and unintended pregnancy. Our findings highlight the need for interventions and service delivery programs tailored for residents in the border region. Further, the study findings were utilized to develop the Sex Ed curriculum and content (e.g., what is consent, gender identity, type of birth control, etc.) for the mobile app. Additionally, we conducted a geo-mapping of exisiting SRH confidential and free services available for cisgender, heterosexual and SGM adolescents in the region including hours of operation, how to qualify for Family planning, access, care and treatment (PACT), scheduling appointmets via the app, etc. Currently the app is being pilot tested for refinment and subsequent larger randomized control trial (RCT).

## Data Availability

The datasets used and/or analysed during the current study are available from the corresponding author on reasonable request.

## Abbreviations

U.S.: United States
STIs: Sexually transmitted infections
AA: African American
SRH: Sexual and reproductive health,SGM: sexual and gender minority
LGBTQIA +: Lesbian, Gay, Bisexual, Transgender, Queer, Intersex, and Asexual
LMFT: Licensed marriage and Family Therapist
PI: Principal investigator
NGOs: Non-government organizations
IDI: In-depth interviews
FGs: Focus groups
Sex Ed: Sexual education
IRB: Institutional Review Boards
PACT: Family planning, access, care, treatment
HRT: Hormone replacement therapy
HPV: Human papillomavirus
